# Structural Brain Correlates of Speech Disfluency in Early Childhood: A Dimensional Analysis in a Population‐Based Cohort

**DOI:** 10.1111/ejn.70652

**Published:** 2026-08-17

**Authors:** Ashmeet Jolly, Aura Yli‐Savola, Elmo P. Pulli, Essi Saloranta, Henry Railo, Harri Merisaari, Ekaterina Saukko, Eero Silver, Venla Kumpulainen, Anni Copeland, Hasse Karlsson, Linnea Karlsson, Niina Junttila, Elina Mainela‐Arnold, Jetro J. Tuulari

**Affiliations:** ^1^ FinnBrain Birth Cohort Study, Turku Brain and Mind Centre, Department of Clinical Medicine University of Turku and Turku University Hospital Turku Finland; ^2^ Department of Psychology and Speech‐Language Pathology University of Turku Turku Finland; ^3^ Department of Teacher Education University of Turku Turku Finland; ^4^ Centre for Population Health Research Turku University Hospital and University of Turku Turku Finland; ^5^ Centre of Excellence in Learning Dynamics and Intervention Research (InterLearn), University of Jyväskylä and University of Turku Finland; ^6^ Department of Radiology Turku University Hospital and University of Turku Turku Finland; ^7^ Clinical Neurosciences University of Turku Turku Finland; ^8^ Department of Psychiatry University of Turku and Turku University Hospital Turku Finland; ^9^ Department of Child Psychiatry University of Turku and Turku University Hospital Turku Finland; ^10^ Department of Public Health University of Turku and Turku University Hospital Turku Finland; ^11^ Neurocenter, Turku University Hospital Turku Finland

**Keywords:** brain structure, grey matter, magnetic resonance imaging, speech disfluency, voxel‐based morphometry

## Abstract

Most neuroimaging studies of speech disfluency have compared individuals who stutter with fluent controls. However, treating speech disfluency as a continuous, dimensional trait offers new insights into the neural basis of fluency during early childhood. We investigated whether naturally occurring variation in speech disfluency is associated with grey matter structure in a population‐based sample of 5‐year‐old children (*N* = 120; 65 M, 55 F). Disfluency was derived from audio and video‐recorded conversational speech samples transcribed and coded using the Ambrose and Yairi classification into stuttering‐like disfluencies (SLDs) and other disfluencies (ODs). T1‐weighted magnetic resonance imaging data were analyzed using voxel‐based morphometry and complemented by surface‐based measures of cortical thickness and surface area (FreeSurfer). Whole brain voxel‐wise regression tested associations between grey matter and disfluency while adjusting for age, sex, handedness, and maternal education, with additional robustness checks (including intracranial volume adjustment and alternative SLD operationalizations). Higher SLDs were positively correlated with regional grey matter volume primarily in the frontal cortex, with clusters in the left middle frontal gyrus and right superior frontal gyrus showing the greatest cross‐model stability (uncorrected voxel‐level *p* < 0.001; FDR‐corrected cluster‐level *p* < 0.05); additional regions, including posterior cerebellum, were more sensitive to model specification. No significant voxel‐wise associations were observed for ODs, and surface‐based analyses yielded no significant associations with SLDs. Boys showed higher SLD rates than girls, but voxel‐wise sex × SLD interactions were not significant. These findings suggest that dimensionally measured SLDs across a population‐based cohort are most consistently related to frontal control systems implicated in planning and monitoring demands during speech. The overlap with frontal regions reported in diagnosis‐based stuttering studies suggests that frontal control circuitry is relevant to fluency variation in early childhood across both dimensional and categorical approaches. These findings support a complementary, dimensional approach to studying fluency variation in early childhood, while underscoring the need for longitudinal work to determine how naturally occurring disfluency relates to later developmental stuttering.

## Graphical abstract

FinnBrain 5‐year‐olds (*N* = 120): speech disfluency measured continuously (SLDs/ODs) from conversational samples. Whole‐brain VBM tested grey matter–disfluency links. Higher SLDs showed robust positive GMV associations in left middle frontal gyrus and right superior frontal gyrus across model checks; ODs and cortical thickness/surface area showed no effects. Frontal control circuitry may support fluency variation, complementing diagnosis‐based stuttering research.

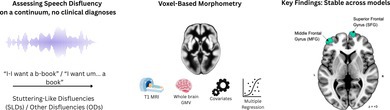

**Abbreviations**
AAL: Automated Anatomical Labeling
CT: cortical thickness
DIVA: Directions Into Velocities of Articulators
DLPFC: dorsolateral prefrontal cortex
eTIV: estimated Total Intracranial Volume
FDR: False Discovery Rate
FOV: Field Of View
FWHM: full‐width at half‐maximum
GMV: grey matter volume
GODIVA: Gradient Order Directions Into Velocities of Articulators
GRAPPA: Generalized Autocalibrating Partially Parallel Acquisition
MFG: middle frontal gyrus
MNI: Montreal Neurological Institute
MRI: Magnetic Resonance Imaging
ODs: other disfluencies
PC9: lobule IX of the left posterior cerebellum
SA: surface area
SALT: Systematic Analysis of Language Transcripts
SFG: superior frontal gyrus
SLDs: stuttering‐like disfluencies
SLP: speech and language pathologist
STROBE: Strengthening the Reporting of Observational Studies in Epidemiology
TE: Echo Time
TI: Inversion Time
TR: Repetition Time
VBM: Voxel‐Based Morphometry

## 1 Introduction

Fluency comprises the seamless flow, pace, and ease of speech production (Schmidt 1992). A speech disfluency refers to interruptions, irregularities, or non‐lexical sounds that disrupt the flow of otherwise fluent speech (ASHA 2024). These may include “false starts,” where words or utterances are abruptly cut off; phrases or syllables that are repeated or restarted; and “fillers,” such as grunts or semi‐articulated sounds like “uh,” “erm,” “um,” or “hmm.”

Occasional disfluency is a natural part of speaking for everyone. During early childhood, speech disfluencies are not only common but often increase as part of typical speech and language development. Normative studies in English‐speaking children show that a range of disfluency types is observed in children who do not stutter, with considerable individual variability in frequency and type (Ambrose and Yairi 1999; Tumanova et al. 2014). Similar findings have been reported in Finnish‐speaking children, where both more typical disfluencies (such as filled pauses and phrase repetitions) and less typical disruptions occur within the context of otherwise typical development (Jansson‐Verkasalo et al. 2021). At the same time, children who go on to be diagnosed with stuttering tend to produce higher rates of certain disfluency types and a different overall profile of disfluencies compared to their typically fluent peers, even relatively early after onset (Ambrose and Yairi 1999; Tumanova et al. 2014). These types of disfluencies may include repeating parts of words (repetitions), lengthening a sound for an extended duration (prolongations), or struggling to begin a word (blocks) (ASHA 2024).

Stuttering‐Like Disfluencies (SLDs) and Other Disfluencies (ODs) are two primary classifications that have been instrumental in both research and clinical settings to diagnose stuttering. In a landmark study, which focused on stuttering in children aged 2–5 years (Ambrose and Yairi 1999), these distinctions were defined based on the nature and frequency of disfluencies (Table 1). Subsequent studies reinforced the utility of this classification, with the criterion of > 3 SLDs per 100 words/syllables emerging as a viable standard threshold for distinguishing between children who stutter and children who do not stutter (Conture 2001; Jansson‐Verkasalo et al. 2021; Yairi and Ambrose 2005). This criterion has demonstrated high sensitivity and specificity across several languages, including Dutch, German, Spanish, and English (Ambrose and Yairi 1999; Boey et al. 2007; Carlo and Watson 2003; Natke et al. 2006). While ODs often occur in fluent speech, they may also appear in the speech of people who stutter, albeit with less frequency and prominence compared to SLDs (Tumanova et al. 2014).

**TABLE 1:** Classification of speech disfluencies (Ambrose and Yairi 1999; Jansson‐Verkasalo et al. 2021).

Stuttering‐Like Disfluencies		
	Types	Description
	Part word repetition	Repetition of a sound or syllable within or in the beginning of the word
	Monosyllabic word repetition	Repetition of a monosyllabic word
	Prolongation	Prolongation of a sound resulting in an unusual duration of that sound
	Block	Stopping airflow and sound during or before production of a vowel or consonant
	Broken words	Stopping of airflow in the middle of a vowel within a word

Other Disfluencies		
	Filled Pause	Filler words or non‐linguistic sounds within an utterance
	Revision and abandoned sentences	Correction of word choice/grammatical or phonological errors/add or delete lexical information
	Multisyllable word repetition	Repetition of a multi‐syllabic word
	Phrase repetition	Repetition of a phrase within an utterance

Some researchers have argued that fluency behaviors may not be well captured by strict binary classifications, and that disfluency can be viewed dimensionally (Adams and Runyan 1981). Consistent with this, stuttering behaviors show substantial within‐person variability across time and situations (Tichenor and Yaruss 2021). In parallel, multifactorial and spectrum‐based perspectives emphasize that stuttering is heterogeneous, with complex interactions among contributing factors and potentially diverse etiologies (SheikhBahaei et al. 2023; Smith and Weber 2017). Stuttering and disfluency may exist along a continuum, reflecting complex interactions between motor, cognitive, linguistic, and emotional factors. This highlights the importance of considering speech disfluency as a spectrum rather than relying solely on binary classification. Fundamentally, even if stuttering ultimately reflects a categorical difference, where individuals who stutter exhibit brain abnormalities distinct from those underlying typical disfluencies, understanding the neural correlates of normal disfluency is essential for identifying what may be unique to clinical stuttering. Conversely, if speech disfluency truly lies on a continuum, then neural correlates of typical disfluency may show partial overlap with regions implicated in clinical stuttering, without implying a single mechanism across the full range. Thus, examining natural variation in speech disfluency provides critical insights for both theoretical models and clinical understanding of fluency disorders.

Neuroimaging studies of stuttering have identified converging abnormalities across functional connectivity, white and grey‐matter brain structure, particularly within speech–motor and auditory brain areas (Etchell et al. 2018). Within this broader multimodal picture, a subset of work has focused specifically on grey matter morphology, reporting structural differences in regions implicated in speech and motor control.

To situate our findings within this literature, we compiled an overview of previous investigations that examined grey matter morphology in children and adults who stutter using voxel‐based morphometry (VBM) (Ashburner and Friston 2000) and/or FreeSurfer (Fischl 2012) for analysis (Table 2). VBM is a whole‐brain, unbiased technique that enables voxel‐wise estimation and statistical comparison of local grey matter volume (GMV), and it has been widely applied in both clinical and developmental samples to detect regional structural differences across the cortex and subcortex (Kurth et al. 2015; Mechelli et al. 2005). Surface‐based measures such as cortical thickness and surface area, as implemented in FreeSurfer, offer a complementary, anatomically anchored perspective: automated methods can estimate thickness across the cortex with submillimeter reliability (Fischl and Dale 2000; Han et al. 2006). Together, these approaches allow us to examine whether disfluency relates primarily to regional variation in GMV, to cortical thickness or surface area, or to some combination of these structural features.

**TABLE 2:** Overview of neuroimaging studies (2000–2025) investigating structural grey matter differences in stuttering.

Study	Technique (Software)	Sample and Age (mean age in years)	Disfluency/Stuttering assessment method used	Key results
Jäncke et al. (2004)	VBM (SPM99)	10 AWS 10 ANS (Mean age = NA)	SSI	AWS vs. ANS: no significant GMV differences (whole brain).
Beal et al. (2007)	VBM (SPM5)	26 AWS (Mean age = 30.29); 28 ANS (Mean age = 30.53)	SSI	AWS > ANS: Higher GMV in L IFG and bilat STG/PT. AWS < ANS: none (no significant decreases).
Chang et al. (2008)	VBM (FSL)	8 persistent CWS (Mean Age = 11) 7 recovered CWS (Mean age = 10.8) 7 CNS (Mean age = 10.7)	SLDs and ODs criteria	CWS < controls: Lower GMV in a L‐lateralized speech–motor/language network. Within CWS (recovered vs. persistent): Recovered < persistent: Lower GMV in L MFG. Within CWS (persistent vs. recovered): Persistent > recovered: Higher GMV across frontal, temporal, motor, parietal, and cerebellar regions.
Kell et al. (2009)	VBM (SPM5)	13 persistent AWS (Mean age = 27); 13 recovered AWS (Mean age = 40); 13 ANS (Mean age = 30)	Boberg and Kully (1994) criteria	Persistent AWS < fluent controls: Lower GMV in L IFG. Recovered AWS < fluent controls: Lower GMV in L IFG. Within persistent AWS: stuttering severity negatively correlated with L IFG GMV (i.e., higher severity → lower GMV). Within recovered AWS: no significant severity–GMV association reported.
Lu et al. (2010)	VBM (SPM5)	10 AWS (Mean age = 24.5); 12 ANS (Mean age = 24)	SSI	AWS > ANS: Higher GMV in L putamen. AWS < ANS: Lower GMV in L medial frontal gyrus and L anterior STG.
Beal et al. (2013)	VBM (SPM8, VBM8, TOM8)	11 CWS (Mean age = 18.07) 11 CNS (Mean age = 22.46)	SSI	CWS < controls: Lower GMV in bilat IFG and L putamen. CWS > controls: Higher GMV in R RO, R MFG, R STG, and R IPL. Within CWS: stuttering severity negatively correlated with GMV in R IFG/RO (higher severity → lower GMV).
Sowman et al. (2017)	VBM (SPM8)	27 AWS (Mean age = 45.9) 27 ANS (Mean age = 47.1)	Self‐report	AWS < ANS: Lower GMV in L caudate nucleus.
Garnett et al. (2018)	FreeSurfer	11 recovered CWS (Mean age = 5.8) 25 persistent CWS (Mean age = 6.4) 34 CNS (Mean age = 6.6)	SSI	Persistent CWS vs. recovered CWS & controls: Lower CT in L vMC, L PMC, and L aCO. Recovered CWS: altered gyrification in medial PMC.
Koenraads et al. (2019)	FreeSurfer	26 CWS (Mean age = 8.18) 489 CNS (Mean age = 7.98)	Parental reports	Children with history of stuttering < fluent peers: Lower GMV in L IFG and SMA. Exploratory surface‐based: Lower CT in L IFG and bilat frontal/parietal regions.
Chow et al. (2023)	VBM (CAT12)	23 recovered CWS (Mean age = 5.1) 72 persistent CWS (Mean age = 5.8) 95 CNS (Mean age = 5.7)	SSI	CWS vs. controls: Lower GMV in putamen, NAc, and thalamus (speech–motor/subcortical structures). Age‐related pattern (within CWS): younger children show primarily putamen reductions
Shojaeilangari et al. (2025)	VBM (CAT12)	15 PDS (Mean age = 27.8), 15 control group (Mean age = 25.66)	SSI	PDS > controls: Higher GM in R postcentral gyrus and L MTG. PDS < controls: Lower GM in L SFG, paracentral lobule, R cuneus, and R cerebellum.

Abbreviations: aCO = anterior central operculum, ANS—Adults who do not stutter, AWS—Adults who stutter, bilat = bilateral, CAT12 = Computational Anatomy Toolbox (CAT12) for SPM; toolbox for structural MRI morphometry (e.g., VBM), CNS—Children who do not stutter, CT = cortical thickness, CWS—Children who stutter, GMV—Grey Matter Volume, IFG = inferior frontal gyrus, IPL = inferior parietal lobule, L = Left, MFG = Middle Frontal Gyrus, MTG = middle temporal gyrus, NAc = nucleus accumbens, PDS = Persistent Developmental Stuttering, PMC = premotor cortex, PT = planum temporale, R = Right, RO = Rolandic operculum, SFG = superior frontal gyrus, SMA = supplementary motor area, SPM8 = Statistical Parametric Mapping (MATLAB‐based software for preprocessing and statistical analysis of neuroimaging data), SSI—Stuttering Severity Instrument, STG = superior temporal gyrus, TOM8 = Template‐O‐Matic (TOM8) toolbox for SPM8; used to generate customized pediatric tissue probability maps (TPMs) for segmentation/normalization in pediatric VBM workflows, VBM—Voxel Based Morphometry, vMC = ventral motor cortex.

By concentrating on these two methods, studies included in Table 2 were identified by selecting relevant VBM‐ and FreeSurfer‐based grey matter reports from the systematic review by Etchell et al. (2018) and by adding more recent work via a PubMed search (keywords: “stuttering,” “voxel‐based morphometry,” “FreeSurfer,” “grey matter”). All these investigations relied on clinical samples (diagnosed persistent or recovered stuttering), as no previous structural neuroimaging work, to our knowledge, has examined subclinical or continuous variation in speech fluency.

The studies summarized in Table 2 indicate that structural neuroimaging work on stuttering has repeatedly identified differences in regions crucial for speech and motor control. Across VBM studies, the left inferior frontal gyrus and superior temporal regions have been frequently implicated, along with basal ganglia structures such as the putamen and caudate nucleus and motor/premotor cortices (Beal et al. 2007, 2013; Chang et al. 2008; Chow et al. 2023; Kell et al. 2009; Lu et al. 2010; Shojaeilangari et al. 2025; Sowman et al. 2017). Surface‐based studies further report reduced cortical thickness in left frontal and motor regions in children who stutter (Garnett et al. 2018; Koenraads et al. 2019). Although directionality (increase or decrease in grey matter) varies, these regions are recurrently implicated in both child and adult studies, suggesting core neural circuits relevant to persistent stuttering.

Contemporary neurocomputational accounts of fluent speech and speech disfluency, particularly DIVA (Directions Into Velocities of Articulators) and its extension GODIVA (Gradient Order DIVA), propose that stable production depends on coordinated interactions among cortical and subcortical speech motor systems (Bohland et al. 2010; Tourville and Guenther 2011). Within this framework, GODIVA specifically describes how cortico‐basal ganglia‐thalamo‐cortical circuits support the selection, sequencing, and initiation of planned speech motor programs. In particular, GODIVA describes how medial frontal speech initiation regions (e.g., the pre‐supplementary and supplementary motor areas) interact with basal ganglia gating mechanisms to enable the orderly progression of speech sequences (Civier et al. 2013; Guenther 2016). From this perspective, SLDs, which reflect breakdowns in speech motor sequencing/initiating and execution, may relate to structural variability in components of these motor sequencing and control circuits. Consistent with a dimensional approach, we focus on individual differences in fluency within a typically developing cohort.

In contrast, ODs are often interpreted as reflecting linguistic formulation and repair processes during connected speech (e.g., lexical selection/retrieval, syntactic planning, revisions, and interjections). These functions map naturally onto the dual‐stream model of speech and language processing (Hickok and Poeppel 2007). In this framework, a ventral stream centered on superior and middle temporal regions supports speech comprehension and lexical–semantic processing, whereas a dorsal stream links posterior temporal–parietal regions with frontal articulatory networks to support phonological–articulatory mapping and sensorimotor integration (Hickok and Poeppel 2007, 2015). Individual differences in OD rates might therefore be expected to relate more to variability in language‐network regions (particularly ventral temporal systems supporting lexical–semantic processing and formulation demands) than to classic speech motor control regions.

Our study builds on previous neuroimaging research by incorporating established methodologies while introducing a dimensional approach to speech fluency: we model disfluency as a continuous score rather than assigning children to categorical stuttering groups. This choice aligns with recent work emphasizing that overt stuttering behaviors fluctuate substantially across days, situations, and individuals, in both adults and young children (Constantino et al. 2016; Hendrick et al. 2023; Rasoli Jokar et al. 2025; Tichenor and Yaruss 2021). Because SLDs and ODs can vary even within the same person, treating disfluency as a continuous trait in a population‐based sample offers a way to capture underlying tendencies in fluency without imposing a rigid diagnostic cutoff.

In this study, we focus on 5‐year‐old children, an age that represents a critical developmental window for speech fluency. Developmental stuttering most often begins between 2 and 4 years of age, and the risk of newly developed stuttering decreases substantially after about age 4–5 (Yairi and Ambrose 2005). By around 5 years of age, children's core speech and language abilities are well established and speech is typically highly intelligible to listeners, although disfluencies remain common in typical development and fluctuate during early childhood (Jansson‐Verkasalo et al. 2021; NIDCD 2017). This age therefore represents a clinically and theoretically important period: in longitudinal studies of early childhood stuttering, many children show natural recovery over the years following onset, whereas others follow a trajectory toward persistence (Yairi and Ambrose 2013, 2005). From a neurodevelopmental perspective, the years around age five are also characterized by rapid but regionally differentiated structural maturation, with longitudinal work showing marked, spatially patterned changes in cortical thickness and grey‐matter development across frontal, temporal, and parietal regions (Gogtay et al. 2004; Sowell et al. 2004).

We conducted whole‐brain analyses to examine the relationship between GMV and disfluency, alongside complementary region‐of‐interest analyses focused on cortical thickness and surface area in five‐year‐old children. Guided by theoretical models and the prior structural literature summarized in Table 2, we expected to find associations in frontal and motor‐control networks for SLDs, and in regions supporting language processing for ODs; however, given the exploratory nature of voxel‐wise analyses, we did not specify a priori hypotheses about the exact location or direction of effects.

## 2 Methods

This study adhered to the principles outlined in the Declaration of Helsinki and received approval from the Joint Ethics Committee of the University of Turku and the Hospital District of Southwest Finland. Approval was granted under two protocols: (1) ETMK: 63/1801/2017 for speech assessments and (2) ETMK: 31/180/2011 for neuroimaging. The parents of the participants were informed about the visits and were requested to explain these to their child in a way that the child could understand. Consent from the child was also obtained.

The reporting was conducted following the STROBE (Strengthening the Reporting of Observational Studies in Epidemiology) guidelines to ensure transparent and comprehensive reporting of our research.

### 2.1 Participants

**FIGURE 1:**
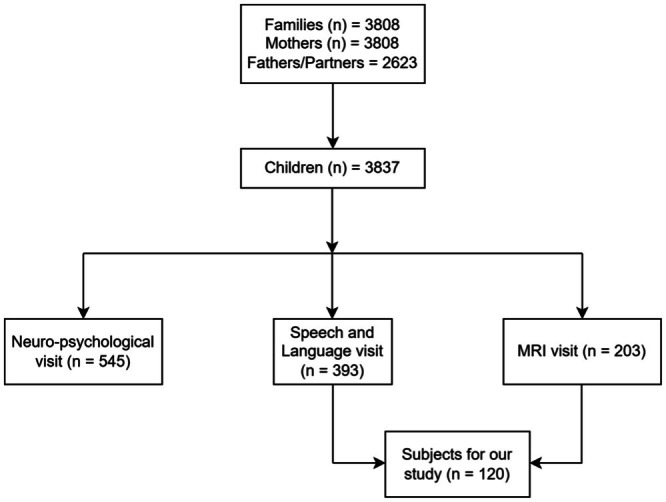
Flowchart of the participants in the current study (the number of children includes 29 twin pairs).

Participants were drawn from the prospective FinnBrain Birth Cohort study that began in 2010 and was established to study the effects of prenatal stress and early life stress on early childhood development from fetal life onwards. After birth, these children took part in longitudinal assessments entailing infant and early childhood brain and cognition (see Figure 1). An overview of the study and the population has been described previously (Karlsson et al. 2018). A total of 120 (65 male, 55 female) Finnish‐speaking children successfully completed the speech assessment and structural neuroimaging visit suitable for the current study (please refer to Table 3 for demographic details).

**TABLE 3:** Demographics of the study population (*n* = 120).

Measure	Mean	SD	Min	Max
*Child Characteristics*				
**Age at scan** *(in months)*				
Boys	65	1.7	62.8	69.4
Girls	64.9	1.3	63	69.1
**Gestational Age at birth** *(in weeks)*				
Boys	39.7	1.7	33.9	42.3
Girls	40.1	1.3	36.3	42.3
**Birth weight** *(in kg)*				
Boys	3.6	0.47	2.53	4.98
Girls	3.52	0.47	2.58	4.9
**Head circumference at birth** *(in cm)*				
Boys	35.48	1.39	32	39
Girls	34.72	1.46	32	38.5
**Handedness**	**Right‐Handed**	**Left‐Handed**	**Ambidextrous**	**Unknown**
Boys	60	1	4	0
Girls	49	5	0	1
*Maternal Characteristics*				
**Age at birth** *(in years)*	31	5	20	41
**BMI at birth** *(in kg/m* ^*2*^ *)*	24.17	4.19	17.48	41.95
**Income** *(estimated after taxes, in euros)*	**Number**		**Percent**	
≤ 1500	33		27.5	
1500–2500	66		55	
2501–3500	13		10.8	
≥ 3501	3		2.5	
Missing	5		4.2	
**Education**				
Low	27		22.5	
Middle	31		25.8	
High	58		48.3	
Missing	4		3.3	
**Background**				
Finnish	115		95.8	
Other	1		0.8	
Missing	4		3.3	
**Alcohol use during pregnancy**				
Yes	20		16.6	
No	85		70.8	
Only before learning about pregnancy	15		12.5	
**Smoking during pregnancy**				
Yes	3		2.5	
No	113		94.1	
Only before learning about pregnancy	4		3.3	
**Illicit drug use** *(gestational week 34)*				
No	117		97.5	
Missing	3		2.5	

*Note:* BMI—Body Mass Index; Smoking status is based on FinnBrain questionnaires and National Institute for Health and Welfare (www.thl.fi); Maternal education: Low = Upper secondary school or vocational school or lower, Middle = University of applied sciences, High = University; Estimated monthly income; Alcohol use were collected through questionnaires administered during the 14th gestational week.

### 2.2 Speech Assessment Visit

For the speech assessment of the 5‐year‐olds, we recruited families who had also participated in the neuropsychological study visits between 2017 and 2021. After contacting them, 393 children ended up participating in the study. Only Finnish‐speaking families were recruited for the speech and language study. The recruitment was done either via phone calls or emailing the families.

All children were monolingual Finnish speakers determined by asking the parent whether there were any other languages spoken in the family besides Finnish. Only children who heard or used Finnish 80% of the time or more participated.

From the initial pool of 394 children that were willing to participate in our study visit, 393 speech samples were acquired, as one child refused to speak. And out of 393, 325 speech samples were long enough (at least 300 words) for fluency analyses.

Speech samples were collected while the child interacted with the examiner in a free play setting. The examiners were speech‐language pathology master's students supervised by a clinical instructor. They spent approximately 20 min engaging in play with the child and encouraging them to speak by asking open‐ended questions and making remarks about their play. Toward the end of the session, the examiner was instructed to create a bit of pressure on the child to increase the likelihood of disfluencies by asking the child to quickly talk about an exciting event before leaving. The play sessions were audio recorded in a wave format using a Marantz recorder with a 44.1 kHz sampling frequency and 16‐bit resolution, and the video recordings were consulted to resolve ambiguous cases. For disfluency coding and rate calculation, a standardized segment comprising the last ~300 words of each session (counted backward from the end of the recording) was analyzed, thereby consistently including the end‐of‐session pressure‐inducing prompt.

### 2.3 Magnetic Resonance Imaging (MRI) Acquisition

The Siemens Magnetom Skyra fit 3 T scanner with a 20‐element head/neck matrix coil was utilized to scan the participants. The image acquisition process was accelerated using the Generalized Autocalibrating Partially Parallel Acquisition (GRAPPA) technique. As a component of a scan protocol lasting a maximum of 60 min, the MRI data were obtained. The current study obtained high resolution T1‐weighted images with the following sequence parameters for its purposes: repetition time (TR) = 1900 ms, inversion time (TI) = 900 ms, echo time (TE) = 3.26 ms, voxel size = 1.0 × 1.0 × 1.0 mm^3^, flip angle = 9°, and field of view (FOV) 256 × 256 mm^2^. The scans were planned as per recommendations of the FreeSurfer developers (FreeSurfer Morphometry Protocols 2009).

### 2.4 MRI Visit

The target age for the neuroimaging visit was 63–65 months, and due to COVID issues there was a delay of 4–5 months in some of the cases (Pulli et al. 2022). Participants who met any of the following criteria were excluded from the MRI: (1) Participants born before gestational week 32, (2) Participants with abnormalities in development for example in senses or communicative abilities (e.g., deafness, blindness and congenital heart disease), (3) Participants who had an established long‐term medical diagnosis (e.g., autism or epilepsy), (4) Participants with ongoing medical assessments (e.g., a child was referred from a primary care setting to special healthcare), (5) Participants who use medication daily and continuously (e.g., oral medications, inhalants, and topical creams. An exception to this was desmopressin (Minirin)), (6) Participants with a history of head trauma, (7) Gold‐plated metallic ear tubes, and routine MRI contraindications.

The scans were done by a team of four Ph.D. students, a research nurse and two radiographers. The participants were allowed to be awake or asleep during the scans, and a member of the research staff and a parent or guardian were present in the room throughout the entire scan. The participants were provided with earplugs and headphones, and they could watch a movie or TV show of their choice through mirrors that were placed inside the head coil. Foam padding was utilized to ensure the participants' head stability and comfort during the scan.

Of the 203 children who participated in this visit, 146 completed both the neuropsychological and speech‐language assessments. Among these 146, 8 did not undergo MRI scanning and 4 were excluded due to excessive motion artifacts in their T1‐weighted images, resulting in 134 participants with usable MRI data. After MRI quality control (Pulli et al. 2022), 121 T1‐weighted images remained. When these MRI datasets were merged with the speech assessment data, one participant was excluded due to missing or poor‐quality speech data. Therefore, the final sample for the present study included 120 children with both high‐quality MRI and speech‐language data (Figure 1).

### 2.5 Data Pre‐Processing

#### 2.5.1 Speech Disfluency

The audio recordings of speech were transcribed using the SALT software v20.0 (Systematic Analysis of Language Transcripts) (Miller and Iglesias 2020) using high‐quality headphones. The final 300–350 words in each sample were examined for fluency. Words that were unintelligible or stand‐alone affirmations or negations (e.g., yes, no, and okay) were not included in the analysis, but those that were immediately followed by another word or phrase were retained (Ambrose and Yairi 1999).

Disfluencies were then identified and categorized based on a system of disfluency types developed by Ambrose and Yairi (1999) and Jansson‐Verkasalo et al. (2021), with further details provided in Table 4.

**TABLE 4:** Types of disfluencies developed by Ambrose and Yairi (1999) and Jansson‐Verkasalo et al. (2021).

Stuttering‐like disfluencies (SLDs)		
Disfluency type	SALT code	Examples in English (in Finnish)
Sound repetition	FLRSnd	d d dog (k k koira)
Syllable repetition	FLRSyl	dan dan dancing (tan tan tanssia)
Part‐word repetition	FLRSyl	compli‐ complicated (ko‐ koira)
Monosyllabic word repetition	FRLMono	and and and (ja ja ja)
Prolongation	FLP	mmmy bag (mmminun laukku)
Block	FLB	… cat (… kissa)
Broken word	FLBW	sing … ing (lau … laen)

Other types of disfluencies (ODs)		
Filled pauses	FP	I was thinking ummm that one (Ajattelin, että (aaa) tuo)
Multisyllabic word repetition	FLRmulti	University university (Yliopisto yliopisto)
Self‐revision and abandoned sentences	() >	I saw‐heard some noise (Minä näin‐kuulin melua) /I thought > What is this? (Ajattelin > Mikä tämä on?)
Phrase repetitions	()	I want I want to run (Haluan Haluan joutsa)

Disfluency types were computed using the per 100 words metric. Given the nature of our speech assessments which were focused on conversational speech, we considered a word‐based measure more appropriate than the syllable‐based metric. Computation of SLDs included combining part word repetition, single‐syllable word repetition, and dysrhythmic phonation, divided by the word count. For ODs, filled pauses, revision/abandoned utterances, and multi‐syllable/phrase repetition were combined and then divided by the word count (for a detailed version of these clauses, please refer to Table 1).

**FIGURE 2:**
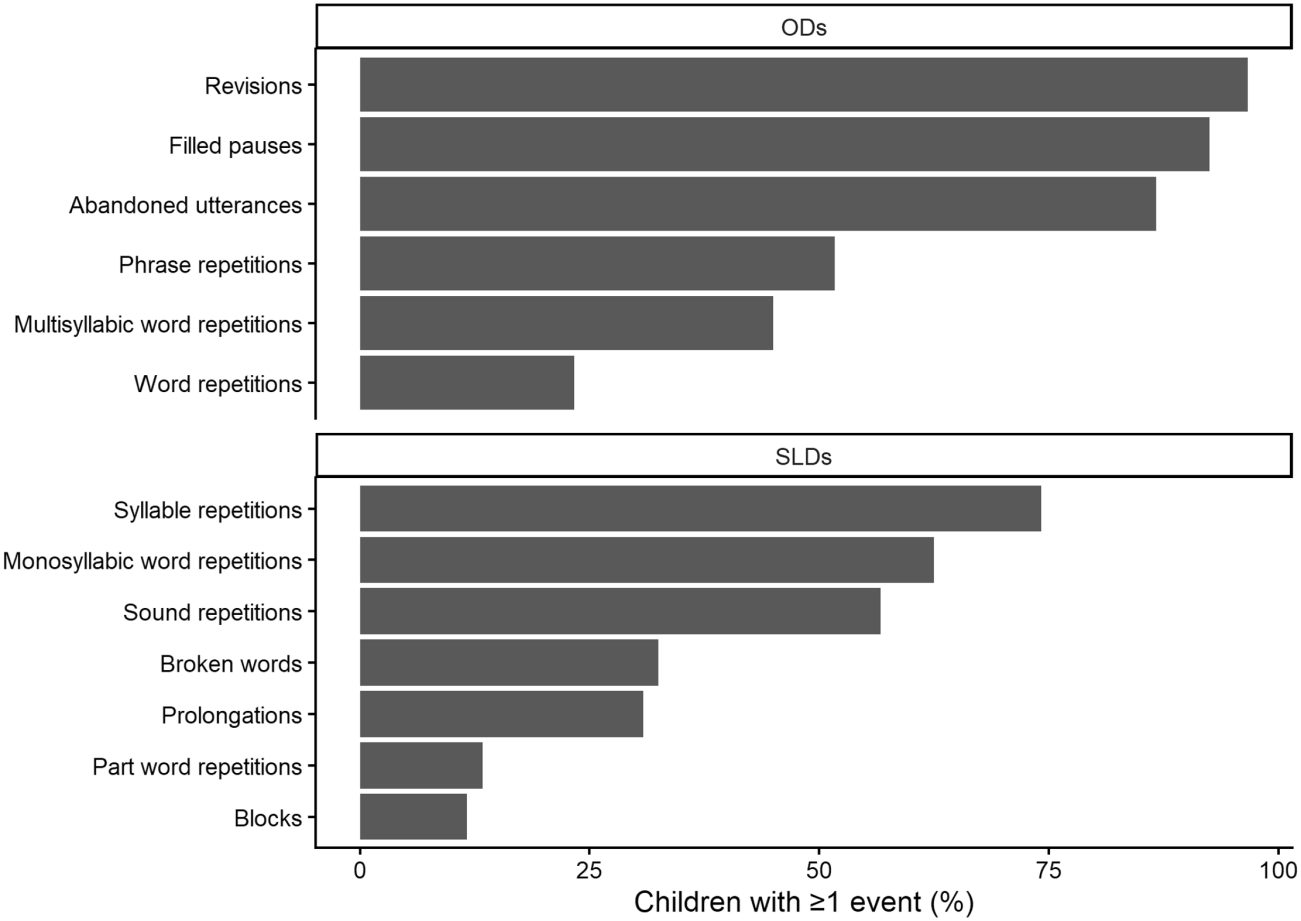
Prevalence of disfluency subtypes across children in the full cohort (*N* = 120). Bars show, for each subtype, the percentage of children who produced at least one instance of that subtype in the speech sample (i.e., subtype count > 0 for that child). Subtypes are shown separately for other disfluencies (ODs; top panel) and stuttering‐like disfluencies (SLDs; bottom panel). Percentages are calculated relative to the full cohort size (*N* = 120); subtypes are not mutually exclusive (a child may contribute to multiple subtypes).

SLDs and ODs were analyzed as a composite rate (aggregate SLD and OD types per 100 words) following the Ambrose & Yairi classification framework, consistent with common practice in developmental fluency research. Because individual SLDs and ODs sub‐types were infrequent in this population‐based cohort, subtype‐specific analyses were not pursued (Figure 2). For robustness, SLDs and ODs were additionally computed per 100 syllables.

The transcriptions were carried out by a Speech and Language Pathologist (SLP) specialized in fluency disorders together with two master's level Speech and Language Pathology students. Students were educated in fluency coding and divided the coding of the samples between them. Another SLP clinically specialized in fluency disorders was treated as the “golden standard” coder who independently re‐coded at least 10% of each coder's (SLP + two students) transcripts. Point‐by‐point reliabilities, along with Cohen's kappa (κ), for location were: SLP (κ = 0.85; point‐by‐point reliability = 0.95), Student 1 (κ = 0.85; point‐by‐point reliability = 0.95), and Student 2 (κ = 0.81; point‐by‐point reliability = 0.95) between the crosschecked transcripts. For type, reliabilities were: SLP (κ = 0.84; point‐by‐point reliability = 0.90), Student 1 (κ = 0.75; point‐by‐point reliability = 0.95), and Student 2 (κ = 0.93; point‐by‐point reliability = 0.96) between the transcripts.

#### 2.5.2 Brain Morphology

VBM was the primary derived brain measure of the current study. Additionally, region‐of‐interest based surface‐based analyses of cortical surface area and cortical thickness assessments were conducted to investigate whether these would complement the VBM findings.

Voxel‐based morphometry was carried out in SPM12 using the CAT12 toolbox (Version r1363) (https://www.fil.ion.ucl.ac.uk/spm/software/). T1‐weighted images were bias‐corrected, segmented into grey matter, white matter, and cerebrospinal fluid using the default SPM tissue probability maps, and spatially normalized to MNI space with CAT12's DARTEL procedure based on the MNI152 template (MNI = Montreal Neurological Institute). CAT12 was set to write modulated, normalized GM images at 1.5 mm isotropic resolution and internal resampling for preprocessing was fixed at 1.0 mm. Modulation rescales voxel intensities by the local Jacobian determinants of the deformation field, so that the resulting images index GMV “corrected” for the amount of warping during spatial normalization. These GMV maps were smoothed using an 8 mm full‐width at half‐maximum (FWHM) Gaussian kernel and entered into the statistical analysis.

The MRI scans were also preprocessed using the FreeSurfer software (version 6.0). Images with artifacts or large unsegmented regions were removed. These scans were manually edited (Pulli et al. 2022). Once the manual quality control had been performed and manual corrections made, the ENIGMA Cortical Quality Control Protocol 2.0 was employed to conduct a quality check (USC, Mark and Mary Stevens Neuroimaging and Informatics Institute, April 2017), after which some regions were excluded. In total, 34 regions of the brain were parcellated (Figure S1).

### 2.6 Statistical Analyses

#### 2.6.1 Whole Brain Voxel‐Wise Analyses

The SLDs and ODs distributions were rightly skewed. To address this, we applied a cube root transformation to normalize the distribution of the data (Figure S1). We then conducted a multiple regression analysis using VBM scans to examine the relationship between SLDs and GMV, including relevant covariates. The covariates examined in this analysis included the mother's level of education, the participant's handedness, age at the time of scanning, and sex of the participant. Parent‐reported family history of stuttering was available for 80 of the 120 children. Of these, 26/80 children had a reported parental history of stuttering. Among the children who exceeded the exploratory > 3 SLDs per 100 syllables screening threshold, only two had a reported parental history of stuttering. Because family‐history data were unavailable for a substantial subset of the cohort, this variable was not included as a covariate in the voxel‐wise models.

Maternal education had three levels: Low = Upper secondary school or vocational school or lower, Middle = University of applied sciences, High = University. To simplify the statistical analysis in current study, the middle and high‐level education of the mother were grouped together, and there were four missing values that were imputed using mode imputation. Handedness, which was measured using parental reports, had two levels: right‐handed and left‐handed, with the four ambidextrous children grouped with right‐handed participants to streamline the analysis. Handedness was included as a covariate in the primary model to maintain consistency with prior research (Koenraads et al. 2019). Sex was categorized into girls and boys. The interaction effect with sex was also examined. Age was incorporated into the statistical model in terms of days. The same analysis was also done separately for ODs. Potential multicollinearity among regressors was assessed using both pairwise correlations among predictors and variance inflation factors from the fitted regression models (Tables S1 and S2).

The analysis was performed in SPM25 using a general linear model (GLM) with “Multiple Regression” on whole‐brain data with a threshold of *p* < 0.001 (one‐tailed), consistent with SPM's contrast‐based testing framework where positive and negative contrasts are defined separately. False Discovery Rate (FDR) corrected cluster level *p*‐values < 0.05 were considered statistically significant. The AAL (Automated Anatomical Labeling) atlas (Tzourio‐Mazoyer et al. 2002) was employed to identify areas exhibiting prominent clusters.

### 2.7 Sensitivity Analysis

To assess whether our VBM findings were robust to (i) adjustment for intracranial volume (ii) alternative operationalizations of SLDs, and (iii) threshold‐based classification, we conducted the following sensitivity analyses:

1. Adjustment for intracranial volume: To address potential confounding by intracranial volume, we additionally repeated the primary VBM model including eTIV (Estimated Total Intracranial Volume) as an additional nuisance covariate.
2. Operationalization of SLDs (words vs. syllables): Because our study models SLDs dimensionally across a population‐based sample (rather than categorically diagnosing stuttering), SLD per 100 words and per 100 syllables are expected to reflect the same underlying between‐child variation in SLD rate. We repeated the voxel‐wise VBM analysis using SLD per 100 syllables to confirm that the findings were not driven by the choice of denominator.
3. Threshold‐based classification: We cannot reliably diagnose clinical stuttering in this cohort based on a single speech sample and a percentage criterion alone; classification would ideally require additional information (e.g., samples from different conversational situations and assessment of concomitant behaviors). However, for an exploratory robustness check approximating a commonly used stuttering classification threshold, we applied the > 3 criterion using SLD per 100 syllables, rather than per 100 words. This choice was motivated by evidence from Finnish‐speaking children indicating that the syllable‐based threshold provides a more appropriate reference for distinguishing typical disfluency from potentially clinically relevant levels in this language context. Specifically, Jansson‐Verkasalo et al. (2021) reported that the > 3 SLD per 100 syllables guideline was applicable across age groups in typically developing Finnish‐speaking children, whereas the > 3 SLD per 100 words guideline was not, implying that a word‐based cutoff would classify a substantial proportion of typically fluent Finnish‐speaking children above the criterion. Under this operational definition, four children would be excluded (see Figure S3).

### 2.8 Complementary Region‐of‐Interest Based Analyses

For surface‐based analysis, we extracted measurements of cortical thickness and surface area from the brain's surface using a tool called aparcstats, which is part of the FreeSurfer software. Desikan‐Killiany Atlas (Desikan et al. 2006) was used to identify the regions of the surface‐based analysis (Pulli et al. 2022). The statistical analysis was performed in R version 4.4.1 and JASP 0.19.3 using Spearman's partial correlations (see Supporting Information).

## 3 Results

### 3.1 Disfluency Type and Sex Differences in Disfluency Scores

As shown in Figure 2, individual disfluency subtypes occurred infrequently in this cohort; therefore, aggregate SLDs and ODs measures were used rather than subtype‐specific outcomes. The correlation between SLDs and ODs in our population was also assessed. The results showed a significant positive correlation between the percentage of SLDs and ODs (*r* (118) = 0.53, *p* < 0.001, 95% CI [0.39, 0.65]), indicating a moderate relationship between the two types of disfluencies. This implied that as SLDs increased, ODs also tended to increase, suggesting a meaningful relationship between these two measures of disfluency in our sample. Boys had significantly higher SLDs values than girls (t (118) = 2.74, *p* = 0.007, 95% CI [0.0517, 0.3204]). Similarly, boys had higher ODs values compared to girls (t (118) = 2.58, *p* = 0.011, 95% CI [0.0323, 0.2451]).

### 3.2 Grey Matter Volume

A significant positive effect was found between SLDs and GMVs in our 5‐year‐old participants (Table 5). This positive association was observed in the left Middle Frontal Gyrus (MFG), lobule IX of the left Posterior Cerebellum (PC9) and the right Superior Frontal Gyrus (SFG). No significant interaction effect of sex was observed in the regression model. There were no significant results for the reverse contrasts testing negative associations between SLDs/ODs and GMV.

**TABLE 5:** Regions of significant clusters with positive association of SLD and GMVs in children; obtained from the whole‐brain VBM analysis.

Region					MNI Coordinate				
	Uncorrected *p*	FDR‐corrected	FWE‐corrected	Laterality	x	y	z	z‐score	Cluster size
Middle Frontal Gyrus	< 0.001	< 0.001	< 0.001	Left	−40	39	18	4.58	1061
Posterior Cerebellum Lobule IX	0.002	0.020	0.045	Left	−16	−51	−54	4.39	349
Superior Frontal Gyrus	< 0.001	0.001	0.001	Right	12	64	4	3.85	725

### 3.3 Surface‐Based Analysis

A complementary surface‐based analysis was conducted using cortical thickness (CT) and surface area (SA) values obtained from FreeSurfer. These metrics were extracted for the regions identified in the VBM analysis, specifically the left MFG and the right SFG. No significant associations were found between SLDs and either cortical thickness or surface area in these regions (left MFG CT: *ρ* = −0.037, *p* = 0.69; left MFG SA: *ρ* = 0.038, *p* = 0.68; right SFG CT: *ρ* = 0.067, *p* = 0.47; right SFG SA: ρ = 0.055, *p* = 0.55). Please refer to the Supporting Information for more information.

### 3.4 Sensitivity Analysis

To evaluate the robustness of the VBM findings, we conducted three sensitivity analyses (Tables S4–S5; Table 6; Figure S5). First, adjusting the primary model for intracranial volume (eTIV) resulted in significant clusters in the left MFG and right SFG. Second, repeating the primary model using SLD per 100 syllables yielded the same pattern of positive associations as the primary word‐based metric, with significant clusters in the left MFG, right SFG, and left PC9. Third, excluding four children meeting the > 3 SLD per 100 syllables criterion altered the spatial pattern of significant clusters (Table 6). In this specification, significant clusters were observed in the left MFG and right SFG, and additional clusters emerged in the left calcarine cortex, postcentral gyrus, and cingulum. In contrast, the cerebellar cluster identified in the primary model was no longer present. Overall, across sensitivity models, frontal associations (left MFG and right SFG) were consistently observed, and additional positive associations emerged in the left calcarine cortex, left middle cingulate cortex, and left postcentral gyrus (Figure S5).

**TABLE 6:** Regions of significant clusters with positive association of SLDs per 100 syllables and GMV after excluding four children.

Region					MNI Coordinate				
	Uncorrected *p*	FDR‐corrected	FWE‐corrected	Laterality	x	y	z	z‐score	Cluster size
Middle frontal gyrus	0.001	0.003	0.008	Left	−40	38	18	4.28	504
Superior frontal gyrus	< 0.000	< 0.000	< 0.000	Right	15	63	12	4.78	1597
Calcarine gyrus	< 0.000	< 0.000	< 0.000	Left	−3	−75	15	4.53	2253
Middle cingulum gyrus	< 0.000	< 0.000	< 0.000	Left	−14	−16	48	3.90	933
Postcentral gyrus	0.001	0.004	0.015	Left	−56	−8	48	3.84	444

## 4 Discussion

We examined speech disfluency dimensionally in a population‐based, typically developing cohort and tested whether individual differences in disfluency were associated with regional grey matter characteristics. In the primary VBM analysis, higher SLDs were associated with GMV in the left MFG, right SFG, and left PC9. Sensitivity analyses indicated that frontal associations (left MFG and right SFG) showed the greatest cross‐model stability, whereas additional clusters, including the cerebellar finding, varied across specifications. Although sequencing/initiating models such as GODIVA motivate an expectation for medial frontal and basal ganglia involvement, we did not observe significant basal ganglia or medial frontal associations here; the present left anterior MFG and right SFG finding therefore aligns more naturally with executive‐control accounts. No significant associations were observed for ODs, suggesting that SLDs and ODs may differ in their neuroanatomical correlates within this sample. Complementary surface‐based analyses of cortical thickness and surface area did not yield significant associations with SLDs.

### 4.1 Type of Speech Disfluency and Sex Differences

By analyzing SLDs and ODs as continuous variables across the full sample, rather than categorizing children as those who stutter versus those who do not, we captured the range of disfluency observed in a typically developing population. This dimensional approach suggests that disfluencies vary gradually rather than occurring exclusively within a clinical subgroup, supporting the view that speech disfluency exists along a continuum (Adams and Runyan 1981). This aligns with previous work showing that children who stutter also produce both stuttering‐like and other (non‐stuttering) disfluencies (Ambrose and Yairi 1999; Johnson 1959; Tumanova et al. 2014; Yairi and Ambrose 2005). The positive association between SLDs and ODs in our sample further supports co‐occurrence of disfluency behaviors. Importantly, describing disfluency as a continuum in this cohort refers to the distribution of behavior and does not, by itself, establish that the same underlying mechanisms operate across the full range of fluency.

At the neuroanatomical level, only SLDs showed significant voxel‐wise associations with grey matter, whereas ODs did not. In the primary VBM analysis, SLDs were associated with grey matter differences in the right SFG, left MFG, and PC9. The left MFG has been implicated in speech monitoring, planning, and executive aspects of verbal fluency (Abrahams et al. 2003; Turken and Dronkers 2011), and the posterior cerebellum has been linked to timing and coordination processes relevant to fluent speech (Ackermann 2008). More broadly, these regions are compatible with speech production frameworks that emphasize control and monitoring demands during fluent articulation (Guenther 2016; Tourville and Guenther 2011). Notably, we did not observe basal ganglia effects often reported in VBM studies of clinical stuttering (e.g., Beal et al. (2013); Lu et al. (2010); Sowman et al. (2017)).

In contrast, the anticipated association between OD and language‐related cortex (inferior frontal, parietal, and temporal regions) was not supported; we found no significant structural links for OD, failing to confirm the hypothesized language‐network involvement from Hickok and Poeppel's (2007) dual‐stream model. Together, these findings suggest that although SLDs and ODs co‐occur, they may not share identical structural correlates in this community sample, underscoring the value of modelling SLDs and ODs separately rather than treating disfluency as a single homogeneous measure.

Our findings also revealed that boys exhibited significantly more disfluencies (both SLDs and ODs) than girls. This finding is consistent with previous research highlighting a higher prevalence of stuttering in boys compared to girls (Proctor et al. 2008). While the current study focuses on speech disfluencies rather than clinically diagnosed stuttering, these results align with broader patterns observed in speech and language development, where boys are often found to experience more challenges than girls (Anastasi 1937; Maccoby 1966; Maccoby and Jacklin 1978). However, it is important to note that the magnitude of sex differences in speech and language development remains debated. While some studies report significant disparities favoring boys (Proctor et al. 2008), others suggest these differences are modest or negligible (Hyde 2005, 2016; Lindberg et al. 2010; Zell et al. 2015). For instance, the developmental trajectory of speech and language abilities may differ across ages, making it challenging to draw definitive conclusions about the long‐term significance of sex differences at any single developmental stage (Etchell et al. 2018).

### 4.2 Grey Matter Association With Speech Disfluency

**FIGURE 3:**
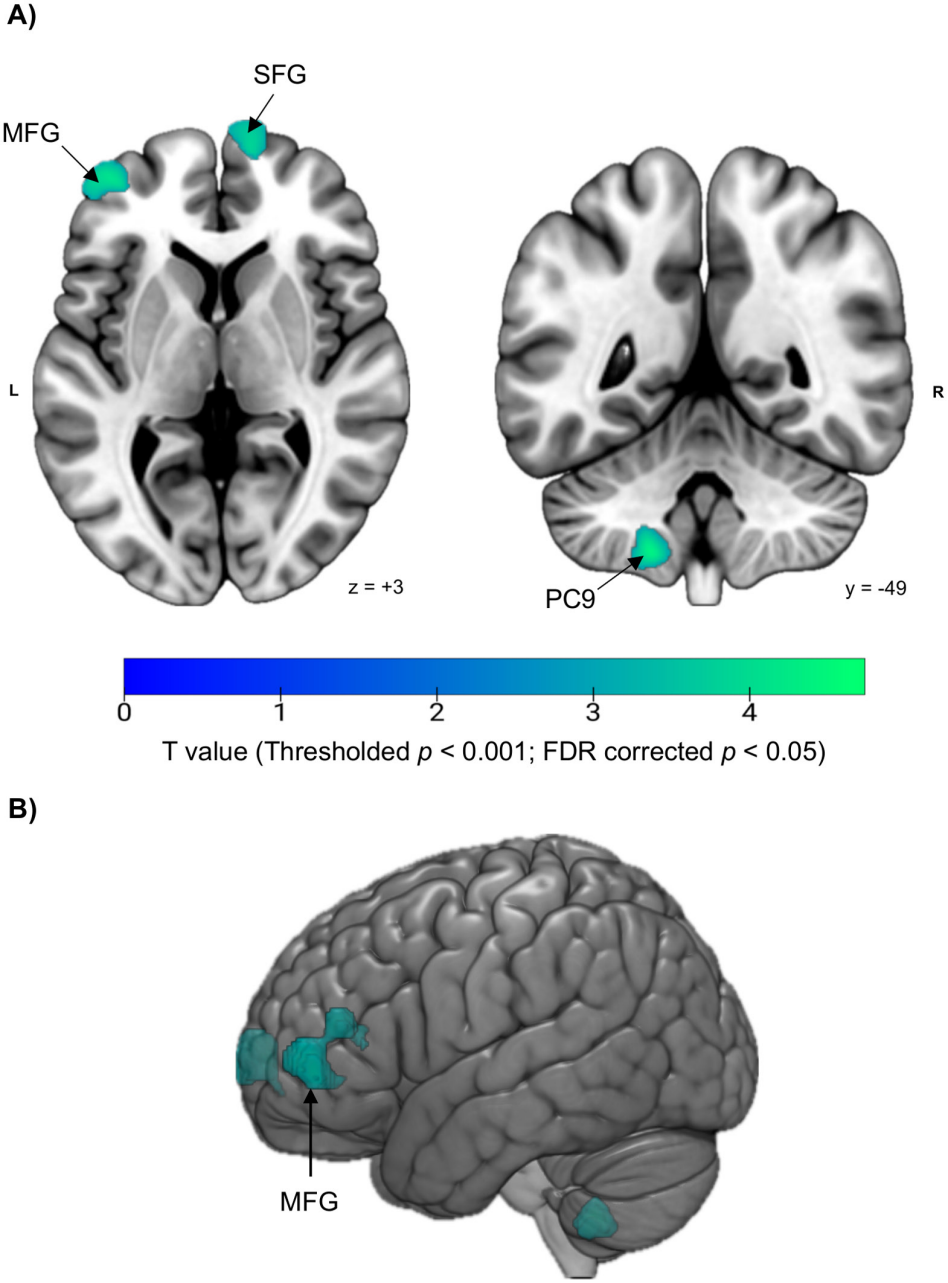
(A) Clusters signify the positive association of SLD with GMV. The color bar represents the T‐scores of the SLD cluster values in the entire population. MFG = Middle Frontal Gyrus; SFG = Superior Frontal Gyrus; PC9 = Posterior Cerebellum Lobule IX. (B) Rendered 3D view of the significant cluster in the left anterior middle frontal gyrus (MFG) associated with higher levels of SLD. The cluster is displayed on the lateral surface of the left hemisphere. This region corresponds approximately to Brodmann area 46.

We found a large cluster in the left anterior MFG, which approximately maps to Brodmann area 46 (BA46) of the dorsolateral prefrontal cortex (DLPFC) (Figure 3B). This falls anatomically outside the classical “Broca's area,” which is confined to the inferior frontal gyrus (BA44 and BA45) (Binkofski and Buccino 2004). BA46 of the anterior MFG is found to subserve domain‐general executive operations like working memory, planning, and cognitive control. These executive functions are relevant to speech fluency because conversational speech requires maintaining and updating an utterance plan and coordinating planning with monitoring, particularly as cognitive demands increase (Hertrich et al. 2021; Ivanova and Ferreira 2019). Consistent with this executive‐support account, behavioral studies report differences in verbal working memory and executive function in children who stutter relative to peers (Kakuta and Kawasaki 2025; Reilly 2005). Experimental work further indicates that increasing concurrent cognitive processing load (dual‐task demands) can affect fluency‐related performance in people who stutter (Bosshardt 2006; Jones et al. 2012).

Though not a core perisylvian language area, the left anterior MFG is often active during language tasks with higher‐order processing (Hertrich et al. 2021). For example, this DLPFC region supports word retrieval in verbal fluency tasks (Abrahams et al. 2003) and the phonological storage component of working memory in speech tasks (Wen et al. 2017). It has also been involved in the broader language network for both language production (e.g., meta‐analytic connectivity studies show BA46 is engaged in the speech production system (Ardila et al. 2016)) and language comprehension (lesion mapping studies in aphasia show BA46 is one of the regions critical for comprehension of language (Turken and Dronkers 2011)).

We also identified a significant cluster in the left posterior cerebellum, specifically in lobule IX. The cerebellum, traditionally recognized for its role in motor coordination, has increasingly been implicated in cognitive functions, including language and speech (E et al. 2014; Hogan et al. 2011; Stoodley and Schmahmann 2009). Research indicates that the posterior cerebellum, particularly lobules VI–IX, is engaged in cognitive processes such as working memory, language, and reading in both adults and children (Grogan et al. 2009; He et al. 2013; Kronbichler et al. 2008; Richardson and Price 2009). Moreover, studies have demonstrated that increased grey matter in these regions is associated with better cognitive performance in children, including vocabulary, reading, and executive functioning (Moore et al. 2017). At the same time, our sensitivity analyses indicated that cerebellar involvement was less stable across model specifications than the frontal findings (Table S4, Table 6), and thus we place greater interpretive weight on the frontal associations.

Building on the involvement of the left MFG in speech disfluency, our analysis also revealed a significant cluster in the right SFG. Right frontal regions are not typically emphasized in classical left‐dominant models of speech production (e.g., left‐biased dorsal stream/fronto–temporal articulatory networks) (Hickok and Poeppel 2007, 2015), but they are frequently discussed in developmental stuttering research in relation to compensatory control, attentional regulation, and altered network dynamics (Neef et al. 2018; Preibisch et al. 2003). In adults who stutter, structural connectivity of right frontal hyperactive areas has been shown to scale with stuttering severity (Neef et al. 2018), and large‐scale neural interaction accounts highlight the contribution of distributed networks, including frontal control regions, to developmental stuttering (Lu et al. 2009). In a typically developing community cohort, right SFG involvement may reflect inter‐individual differences in executive control or monitoring resources that support fluency, rather than a stuttering‐specific neural signature. Notably, the right SFG finding was among the most stable across our sensitivity analyses (Tables S4–S5, Table 6), supporting its relevance as a candidate correlate of SLDs in this sample while still warranting cautious interpretation given the exploratory, voxel‐wise nature of the analyses.

### 4.3 Relation to Prior VBM and Surface‐Based Findings

Table 2 highlights that structural findings in stuttering are heterogeneous across age groups, phenotyping approaches, and analytic pipelines. In this context, our primary voxel‐wise associations with SLD involved frontal cortex (left MFG and right SFG) and posterior cerebellum. The left MFG finding is notable because frontal alterations in this region have also been reported in children who stutter; for example, Chang et al. (2008) identified grey matter differences involving the left MFG in relation to stuttering persistence/recovery, supporting the relevance of frontal executive/control regions for speech fluency. By contrast, direct correspondence for the right SFG finding is less straightforward: while Shojaeilangari et al. (2025) reported a superior frontal region on the left in persistent developmental stuttering, our association was asymmetric and only seen in the right hemisphere, which may reflect differences in phenotype (clinical stuttering vs. continuous disfluency variation), developmental stage, or the possibility that right frontal regions contribute through broader control/monitoring processes rather than speech‐specific representations.

Regarding the cerebellar finding, prior studies have also implicated cerebellar regions, although with variability in laterality and direction. For example, Chang et al. (2008) reported left cerebellar differences in relation to recovery/persistence, and Shojaeilangari et al. (2025) observed reduced grey matter in the right cerebellum in persistent developmental stuttering compared with controls. In our sample, posterior cerebellar involvement was more sensitive to model specification than the frontal clusters. Specifically, the PC9 was not retained in the threshold‐based exclusion analysis (excluding the four children exceeding the > 3% SLD per 100 syllables screening guideline). Given prior reports of cerebellar involvement in developmental stuttering, this pattern is consistent with the possibility that cerebellar associations may be more evident at the upper end of the SLD distribution and potentially among children at higher risk for clinically relevant stuttering. However, because this threshold is used for exploratory screening only and diagnosis/persistence cannot be determined from a single sample at age five, we interpret the cerebellar finding as hypothesis‐generating and focus primarily on the more robust frontal results.

Several VBM studies in clinical stuttering also report basal ganglia involvement, particularly putamen/caudate differences (Beal et al. 2013; Lu et al. 2010; Sowman et al. 2017), which we did not observe. This discrepancy may reflect differences in severity range and assessment (dimensional variation in a community cohort vs. case–control comparison in clinically verified persistent stuttering). Importantly, even if disfluency varies continuously at the behavioral level, overlapping anatomical loci across dimensional and clinical samples should not be assumed to imply identical underlying mechanisms.

We observed positive associations between SLDs and GMV, whereas many diagnosis‐based case–control VBM studies of children who stutter report reduced GMV relative to fluent controls. At the same time, directionality in the stuttering VBM literature is not uniform: both increases and decreases have been reported across studies, populations, and analytic choices, see Table 2 (e.g., adults: Beal et al. 2007, 2013; Lu et al. 2010). Moreover, our analysis tests dimensional variation within a population‐based cohort rather than a diagnosis‐based group contrast, and these designs are not directly comparable. Accordingly, while our findings highlight frontal control circuitry as a robust correlate of SLD variation in early childhood, we avoid inferring mechanistic continuity with clinically diagnosed stuttering solely from regional overlap and emphasize the need for longitudinal follow‐up and richer clinical phenotyping to determine how direction and regional effects relate to later stuttering outcomes.

Finally, our complementary surface‐based analyses of cortical thickness and surface area yielded no significant associations with SLDs. This null result suggests that the observed voxel‐wise associations may reflect properties captured more readily by voxel‐based grey matter measures than by surface‐derived thickness/area in this sample. VBM is sensitive to fine‐grained variations in tissue composition and local density, which may not always manifest in surface‐based measures (Goto et al. 2022). More broadly, Table 2 also indicates that surface‐based findings in stuttering are mixed (Garnett et al. 2018; Koenraads et al. 2019), underscoring that different morphometric approaches may capture partly distinct aspects of neuroanatomy.

### 4.4 Sensitivity Analysis

VBM findings can be sensitive to modelling decisions, particularly in developmental samples where both anatomy and behaviour show substantial inter‐individual variability. We therefore used three robustness checks to clarify what aspects of the SLD–GM pattern are likely to reflect a stable association versus what may be more specification‐dependent.

First, adding eTIV addressed the concern that apparent regional effects might primarily reflect overall head‐size scaling rather than regionally specific variation. The fact that the key frontal pattern persisted after head‐size adjustment makes a global scaling explanation less plausible and supports the interpretation that the frontal findings reflect regional differences beyond overall intracranial volume. Second, repeating the analysis with SLDs computed per 100 syllables addressed whether the observed associations depended on how SLDs were quantified in conversational speech. The similarity of the anatomical pattern across word‐ and syllable‐based operationalizations suggests that the findings are not an artifact of denominator choice, which is important given that the speech sample was conversational and the study was designed to treat disfluency dimensionally rather than categorically. Third, excluding the four children who exceeded the > 3 SLDs per 100 syllables threshold provided a pragmatic check on whether the voxelwise associations were driven by a small number of high‐SLD observations. The persistence of the frontal pattern in this analysis suggests that the main frontal associations are not solely attributable to potential “outliers” at the upper end of the distribution. The additional clusters observed only in this model (calcarine, middle cingulate, and postcentral cortex) were not consistent across sensitivity specifications and are therefore interpreted cautiously. Of these, the postcentral cluster lies in peri‐Rolandic sensorimotor cortex (the pre/postcentral gyri surrounding the central sulcus), a region central to motor–somatosensory processing and has been implicated in stuttering neuroimaging work (Shojaeilangari et al. 2025). The middle cingulate has been linked to cognitive–motor control and performance monitoring, functions that could plausibly relate to speech production demands but are not specific to stuttering (Hoffstaedter et al. 2013). Calcarine cortex corresponds to primary visual cortex (V1) and is commonly engaged during visual/audiovisual speech contexts (Campbell and MacSweeney 2012; Noesselt et al. 2007), but it is not typically interpreted as a speech‐specific region; accordingly, its appearance in the exclusion model is treated as exploratory and nonspecific. This variation in additional regions across specifications reinforces a cautious, hypothesis‐generating interpretation of non‐frontal clusters and highlights the value of future work using repeated speech sampling and richer clinical phenotyping to determine whether these less stable effects reflect smaller true associations or model‐dependent variability.

Taken together, these checks support a graded interpretation of the neuroanatomical findings: the frontal associations represent the most stable candidate correlates of SLDs in our study, whereas other regional effects appear more contingent on analytic specification.

### 4.5 Strengths and Limitations

One of the major strengths of the current study is the dimensional (continuous) modelling of disfluency instead of categorical stuttering diagnosis, preserving information across the full distribution and reducing arbitrary thresholding. We conducted our research on 5‐year‐old participants, which can be considered an appropriate age to identify structural brain anomalies for speech disruptions. We also had a sizeable sample of 120 children with usable speech and neuroimaging data, which could aid future research in exploring the structural similarities and differences in the brains of individuals with speech difficulties. Overall, our study has the potential to contribute to the literature on speech disfluencies.

Several limitations should be noted. First, the cross‐sectional design precludes causal inference regarding whether structural differences precede, follow, or co‐develop with disfluency. Second, VBM findings can be sensitive to modelling choices; accordingly, we conducted sensitivity analyses (including eTIV adjustment, alternative SLD operationalization, and an exploratory exclusion of children exceeding a screening threshold), which suggested that frontal associations were the most stable, whereas other clusters varied across specifications. Third, disfluency was operationalized using SLD and OD composites derived from a single conversational sample. Other approaches (e.g., subtype‐specific associations, SSI‐based severity scoring, clinician diagnosis incorporating concomitant behaviors, repeated samples, or parent‐report measures) capture different dimensions of fluency; therefore, generalization to clinically defined stuttering or alternative fluency constructs should be made with caution. Moreover, stuttering onset and persistence cannot be determined at age five from a single sample, and we cannot specify the potential effects of the children who may later meet criteria for persistent developmental stuttering. Clinical predictors of persistence include age of onset, family history, and disfluency characteristics (Singer et al. 2020); accordingly, we interpret this cohort as population‐based and avoid diagnostic inferences.

## 5 Conclusion

In this exploratory study of 5‐year‐old children, higher SLDs were associated with regional grey matter differences primarily in frontal cortex, with the left MFG and right SFG representing the most robust findings across analytic specifications. Additional associations, including posterior cerebellar involvement, were more sensitive to model specification. No significant voxel‐wise associations were observed for ODs, and complementary surface‐based analyses of cortical thickness and surface area did not yield significant effects. Together, these results suggest that individual differences in SLDs in early childhood are most consistently related to frontal control systems implicated in planning and monitoring demands during speech. Notably, our dimensional approach—without categorizing children as those who stutter and those who do not—identified structural differences in brain regions that have also been implicated in clinical studies of stuttering. Regional convergence with prior stuttering studies suggests frontal control circuitry is relevant to fluency variation in early childhood, but differences in directionality and design mean mechanistic continuity should not be assumed. Our findings support the value of modelling disfluency dimensionally in early childhood, while recognizing that clinical cutoffs remain important for diagnosis; examining the full distribution may help contextualize where thresholds are applied during a developmentally dynamic period when onset and persistence cannot be inferred from a single sample. Replication of our findings with repeated speech sampling, richer clinical phenotyping, and multimodal imaging will be important to clarify the developmental significance and specificity of these associations.

## Author Contributions

**Ashmeet Jolly:** conceptualization, formal analysis, investigation, visualization, methodology, writing – original draft, writing – review and editing, software. **Aura Yli‐Savola:** data curation, writing – review and editing. **Elmo P. Pulli:** data curation, methodology, writing – review and editing. **Essi Saloranta:** data curation, writing – review and editing. **Henry Railo:** writing – original draft, writing – review and editing. **Harri Merisaari:** data curation, writing – review and editing. **Ekaterina Saukko:** data curation, writing – review and editing. **Eero Silver:** data curation, writing – review and editing. **Venla Kumpulainen:** data curation, writing – review and editing. **Anni Copeland:** data curation, writing – review and editing. **Hasse Karlsson:** project administration, writing – review and editing. **Linnea Karlsson:** project administration, writing – review and editing. **Niina Junttila:** funding acquisition, writing – review and editing. **Elina Mainela‐Arnold:** conceptualization, supervision, writing – review and editing, writing – original draft. **Jetro J. Tuulari:** conceptualization, data curation, funding acquisition, methodology, supervision, writing – review and editing, writing – original draft.

## Funding

**AJ**: EDUCA Flagship, Research Council of Finland (#358924, #358947), Ministry of Education and Culture (#VN/3137/2024‐OKM‐4), **EPP**: Strategic Research Council (SRC) established within the Research Council of Finland (#352648 and subproject #352655), Signe and Ane Gyllenberg Foundation, **EsS**: Turku University Graduate School, **HM**: State Research Funding, Turku Centre Hospital, **LK**: Strategic Research Council (SRC) established within the Research Council of Finland (#352648 and subproject #352655), Research Council of Finland #308589, Signe and Ane Gyllenberg Foundation, Finnish State Grants for Clinical Research, **HK**: Stiftelsen Eschnerska Frilasarettet sr, Jane and Aatos Erkko Foundation, **NJ**: EDUCA Flagship, Academy of Finland (#358924 and #358947), **EMA**: Anonymous endowed fund to University of Turku Speech‐Language Pathology, **JJT**: Juho Vainio Foundation; the Hospital District of Southwest Finland, Finnish State Grants for Clinical Research (ERVA); Emil Aaltonen Foundation; Alfred Kordelin Foundation; Sigrid Jusélius Foundation; Signe and Ane Gyllenberg Foundation; the Orion Research Foundation. Open Access funding provided by the University of Turku.

## Conflicts of Interest

The authors declare no conflicts of interest.

## Supporting information


**Figure S1:** Distribution of SLDs and ODs (per 100 words) shown as raw values and cube‐root–transformed values (y‐axis: number of participants). Threshold‐based screening (> 3%) was evaluated separately using syllable‐based SLD rates (see sensitivity analyses and Figure S3).
**Table S1:** Zero‐order correlations among regressors for SLDs.
**Table S2:** Zero‐order correlations among regressors for ODs.
**Figure S2:** (A) Using the Desikan‐Killiany Atlas and the ENIGMA quality control protocol, the 34 parcellated regions are identified and color coded for one of our 5‐year‐old participants; Image Source: USC, Mark and Mary Stevens Neuroimaging and Informatics Institute, April 2017; (B) Color‐coded references for the regions.
**Table S3:** Spearman's partial correlations: cortical thickness and surface area.
**Table S4:** Regions of significant clusters with positive association of SLDs per 100 syllables and GMV after adding eTIV as an additional covariate.
**Table S5:** Regions of significant clusters with positive association of SLDs per 100 syllables and GMV.
**Figure S3:** Distribution of SLDs (per 100 syllables) with the threshold‐based screening (> 3%) illustrated. The blue shaded bars highlight the four children exceeding the syllable‐based screening guideline; this threshold is used for exploratory screening only and does not constitute a clinical diagnosis.
**Figure S4:** SLDs subtype profile in the threshold‐based screen‐positive subset (*N* = 4; > 3% SLDs per 100 syllables). Bars show the total number of events for each SLD subtype pooled across the four children. Labels indicate the number of children (out of 4) who produced at least one instance of the subtype.
**Figure S5:** Sensitivity analyses for voxel‐wise associations between SLDs and GMV. Thresholded statistical maps show positive associations between SLDs and GMV from three alternative model specifications: (A) primary model with intracranial volume included as a nuisance covariate (eTIV), (B) SLDs operationalized as rate per 100 syllables, and (C) SLDs per 100 syllables after excluding children exceeding the > 3% screening guideline (*n* = 4). Maps are displayed in MNI space on the study template; slice coordinates are shown. Color bar indicates T values (voxelwise *p* < 0.001, cluster‐level FDR *p* < 0.05).

## Data Availability

The Finnish law and ethical permissions do not allow open sharing of the data used in this study, but data access is possible via formal material transfer agreements (MTA). Investigators that wish to access the data are encouraged to contact Principal Investigator of the FinnBrain Birth Cohort study Linnea Karlsson (linnea.karlsson@utu.fi).
