# Supplementary material for: Structural Brain Correlates of Speech Disfluency in Early Childhood: A Dimensional Analysis in a Population‐Based Cohort
Source: Eur J Neurosci. 2026 Aug 17;64(4):e70652. doi: 10.1111/ejn.70652 (PMC13481122; doi:10.1111/ejn.70652)
Supplement: Supplementary file 1 [file EJN-64-0-s001.docx]

Supplementary material to

**Structural Brain Correlates of Speech Disfluency in Early Childhood: A Dimensional Analysis in a Population-Based Cohort**

# Ashmeet Jolly^1,2,3,4^✉, Aura Yli-Savola^1,2,4^, Elmo P. Pulli^1,4,5^, Essi Saloranta^1,2,4^, Henry Railo^2^, Harri Merisaari^1,4^, Ekaterina Saukko^6^, Eero Silver^1,4^, Venla Kumpulainen^1,4^, Anni Copeland^1,4^, Hasse Karlsson^1,4,7,8^, Linnea Karlsson^1,4,9,10^, Niina Junttila^3^, Elina Mainela-Arnold^1,2,5^, Jetro J. Tuulari^1,4,5,7,8,11^

^1^FinnBrain Birth Cohort Study, Turku Brain and Mind Centre, Department of Clinical Medicine, University of Turku and Turku University Hospital, Turku, Finland

^2^Department of Psychology and Speech-Language Pathology, University of Turku, Turku, Finland

^3^Department of Teacher Education, University of Turku, Turku, Finland

^4^Centre for Population Health Research, Turku University Hospital and University of Turku, Turku, Finland

^5^Centre of Excellence in Learning Dynamics and Intervention Research (InterLearn), University of Jyväskylä and University of Turku, Finland.

^6^Department of Radiology, Turku University Hospital and University of Turku, Turku, Finland

^7^Clinical Neurosciences, University of Turku, Turku, Finland.

^8^Department of Psychiatry, University of Turku and Turku University Hospital, Turku, Finland

^9^Department of Child Psychiatry, University of Turku and Turku University Hospital, Turku, Finland

^10^Department of Public Health, University of Turku and Turku University Hospital, Turku, Finland

^11^Neurocenter, Turku University Hospital, Turku, Finland.

✉Correspondence: [ashmeet.a.jolly@utu.fi](mailto:ashmeet.a.jolly@utu.fi)

FinnBrain Birth Cohort Study, Turku Brain and Mind Center, Kiinamyllynkatu 10, Medisiina A Building, 20520, Turku, Finland.

**Supplementary Figure S1.** Distribution of SLDs and ODs (per 100 words) shown as raw values and cube-root–transformed values (y-axis: number of participants). Threshold-based screening (>3%) was evaluated separately using syllable-based SLD rates (see Sensitivity Analyses and Supplementary Figure S3).

*Multicollinearity check*

To assess potential multicollinearity among regressors included in the VBM models, we computed pairwise Pearson correlations between disfluency types and all nuisance covariates. We also calculated variance inflation factors (VIFs) for the corresponding multiple regression design matrices.

**Supplementary Table S1**. Zero-order correlations among regressors for SLDs

| Variable |  |  |  |  |  |  |
| --- | --- | --- | --- | --- | --- | --- |
| SLDs | - |  |  |  |  |  |
| Age | -0.06 | - |  |  |  |  |
| Sex | -0.20 | -0.02 | - |  |  |  |
| Handedness | -0.05 | 0.07 | 0.17 | - |  |  |
| Maternal Education | 0.11 | 0.12 | -0.18 | 0.15 | - |  |
| eTIV | 0.23 | 0.00 | -0.59 | -0.14 | 0.18 | - |

**Supplementary Table S2.** Zero-order correlations among regressors for ODs

| Variable |  |  |  |  |  |  |
| --- | --- | --- | --- | --- | --- | --- |
| ODs | - |  |  |  |  |  |
| Age | 0.01 | - |  |  |  |  |
| Sex | -0.24 | -0.02 | - |  |  |  |
| Handedness | -0.04 | 0.07 | 0.17 | - |  |  |
| Maternal Education | 0.14 | 0.12 | -0.18 | 0.15 | - |  |
| eTIV | 0.23 | 0.00 | -0.59 | -0.14 | 0.18 | - |

The largest pairwise correlation was observed between sex and eTIV (|r| = 0.59). Consistent with this, VIFs indicated no problematic multicollinearity (VIF range = 1.02–1.58; max VIF = 1.58).

*FreeSurfer Processing*

FreeSurfer version 6.0.0 (https://surfer.nmr.mgh.harvard.edu/) was used to process T1-weighted brain images of 5-year-old children, performing semiautomated segmentation of cortical metrics such as cortical thickness and surface area. Cortical metrics were extracted using the "aparcstats" function, which computes region-wise statistics based on the Desikan-Killiany atlas. The study employed additional quality control procedures, including manual edits and a dichotomous quality rating system, to address segmentation errors caused by motion artifacts. While manual edits minimally affected cortical thickness results (less than 2%), quality checks were critical for reliable data. For more information, please see (Pulli et al., 2022).

**Supplementary Figure S2**. (A) Using the Desikan-Killiany Atlas and the ENIGMA quality control protocol, the 34 parcellated regions are identified and color coded for one of our 5-year-old participants; Image Source: USC, Mark and Mary Stevens Neuroimaging and Informatics Institute, April 2017; (B) Color-coded references for the regions

*Statistical analysis*

R version 4.4.1 and JASP 0.19.3 were used to perform the statistical analysis. We focused on two regions of interest to explore whether they could complement the VBM findings: the left middle frontal gyrus (MFG) and the right superior frontal gyrus (SFG). Among the parcellated regions, we specifically selected the right SFG and left rostral MFG, as these corresponded to areas where significant clusters were observed. Cortical thickness and surface area measures were extracted for these regions, although some data were missing for certain participants in the study.

Missing data for cortical thickness and surface area measures were addressed using two different imputation methods based on the extent of missingness. For the left rostral MFG, where only four values were missing, mean imputation was performed. This approach was deemed appropriate given the minimal proportion of missing values, ensuring the dataset's integrity without introducing significant bias. For the right SFG, with 31 missing values, multiple imputation using chained equations (MICE) was implemented (Buuren & Groothuis-Oudshoorn, 2011). The MICE procedure employed predictive mean matching (PMM) as the imputation method, incorporating age at scan and sex as predictors to improve estimation accuracy. Five imputed datasets were generated with a maximum of 50 iterations, and the first imputed dataset was selected for analysis. This method provided robust imputed values by leveraging the relationships between cortical thickness/surface area and the chosen predictors while accommodating the higher proportion of missing data.

We replicated the VBM analysis by including the same covariates used in the SPM model. We conducted Spearman’s partial correlation (as these values were not normally distributed) for both cortical thickness and surface area of the left MFG and the right SFG.

**Supplementary Table S3.** Spearman’s partial correlations: cortical thickness and surface area

| **Metric** | **ROI** | **n** | **Spearman’s rho (ρ)** | **p-value** |
| --- | --- | --- | --- | --- |
| Cortical thickness | Left MFG | 120 | -0.037 | 0.696 |
| Cortical thickness | Right SFG | 120 | 0.067 | 0.477 |
| Surface area | Left MFG | 120 | 0.038 | 0.685 |
| Surface area | Right SFG | 120 | 0.055 | 0.556 |

*Note*. Partial Spearman correlations conditioned on sex, mother’s education, age, and handedness. * p < .05, ** p < .01, *** p < .001.

As shown in the results, there is no significant relationship between SLDs and the cortical thickness or the surface area of either the left MFG or the right SFG. This indicates that, once accounting for other variables, there is no statistically meaningful association between these two factors and SLDs for these areas.

*Sensitivity analysis*

1. We repeated the primary voxel-wise multiple regression model (SLD per 100 words; covariates: age, sex, handedness, maternal education) with estimated intracranial volume (eTIV) added as an additional nuisance covariate. All preprocessing and statistical inference settings were identical to the primary analysis (voxel threshold *p* < .001; cluster-level FDR *p* < .05; positive and negative contrasts tested separately). In this model, significant positive associations between SLD per 100 words and GMV remained, whereas no negative associations were observed. Full cluster statistics are shown in Supplementary Table S4 and thresholded maps in Supplementary Figure S5 A.

**Supplementary Table S4**. Regions of significant clusters with positive association of SLDs per 100 syllables and GMV after adding eTIV as an additional covariate.

|  |  |  |  |  | **MNI Coordinate** | | |  |  |
| --- | --- | --- | --- | --- | --- | --- | --- | --- | --- |
| **Region** | **Uncorrected *p*-value** | **FDR-corrected** | **FWE-corrected** | **Laterality** | **x** | **y** | **z** | **z-score** | **Cluster size** |
| **Middle Frontal Gyrus** | <0.000 | 0.004 | 0.014 | Left | -39 | 52 | 4 | 3.96 | 394 |
| **Superior Frontal Gyrus** | <0.000 | 0.004 | 0.015 | Right | 12 | 63 | 3 | 3.66 | 389 |

2. To evaluate whether results depended on the denominator used to compute SLD, we repeated the primary voxel-wise model using SLD per 100 syllables as the predictor of interest. Covariates and inference settings were identical to the primary analysis. This specification yielded significant positive associations between SLD per 100 syllables and GMV in the same anatomical regions as the primary word-based model; no negative associations were detected. Consistent with the primary analysis, no statistically significant associations were observed for ODs. Full results are provided in Supplementary Table S5 and Supplementary Figure S5 B.

**Supplementary Table S5**. Regions of significant clusters with positive association of SLDs per 100 syllables and GMV

|  |  |  |  |  | **MNI Coordinate** | | |  |  |
| --- | --- | --- | --- | --- | --- | --- | --- | --- | --- |
| **Region** | **Uncorrected *p*-value** | **FDR-corrected** | **FWE-corrected** | **Laterality** | **x** | **y** | **z** | **z-score** | **Cluster size** |
| **Middle Frontal Gyrus** | <0.000 | <0.000 | <0.000 | Left | -40 | 39 | 18 | 4.50 | 1106 |
| **Posterior Cerebellum Lobule IX** | <0.000 | 0.005 | 0.013 | Left | -16 | -51 | -54 | 4.55 | 458 |
| **Superior Frontal Gyrus** | <0.000 | 0.001 | 0.001 | Right | 12 | 64 | 4 | 3.78 | 693 |

**Supplementary Figure S3.** Distribution of SLDs (per 100 syllables) with the threshold-based screening (>3%) illustrated. The blue shaded bars highlight the four children exceeding the syllable-based screening guideline; this threshold is used for exploratory screening only and does not constitute a clinical diagnosis.

**Supplementary Figure S4**. SLDs subtype profile in the threshold-based screen-positive subset (N = 4; >3% SLDs per 100 syllables). Bars show the total number of events for each SLD subtype pooled across the four children. Labels indicate the number of children (out of 4) who produced at least one instance of the subtype.

3. As an additional robustness analysis, we repeated the syllable-based SLDs model after excluding four children who met the >3 SLD per 100 syllables criterion. This criterion was used only as an operational definition for this sensitivity analysis, consistent with evidence that syllable-based thresholds provide a more appropriate reference for Finnish-speaking children than word-based thresholds (Jansson-Verkasalo et al., 2021). Under this specification, significant positive associations were observed between SLD per 100 syllables and GMV; no negative associations were detected. Cluster-level results and maps are reported in Table 6 and Supplementary Figure S5 C.

**Supplementary Figure S5.** Sensitivity analyses for voxel-wise associations between SLDs and GMV. Thresholded statistical maps show positive associations between SLDs and GMV from three alternative model specifications: (A) primary model with intracranial volume included as a nuisance covariate (eTIV), (B) SLDs operationalized as rate per 100 syllables, and (C) SLDs per 100 syllables after excluding children exceeding the >3% screening guideline (n=4). Maps are displayed in MNI space on the study template; slice coordinates are shown. Color bar indicates T values (voxelwise p < 0.001, cluster-level FDR p < 0.05).

**References**

Buuren, S. van, & Groothuis-Oudshoorn, K. (2011). mice: Multivariate Imputation by Chained Equations in R. *Journal of Statistical Software*, *45*, 1–67. https://doi.org/10.18637/jss.v045.i03

Pulli, E. P., Silver, E., Kumpulainen, V., Copeland, A., Merisaari, H., Saunavaara, J., Parkkola, R., Lähdesmäki, T., Saukko, E., Nolvi, S., Kataja, E.-L., Korja, R., Karlsson, L., Karlsson, H., & Tuulari, J. J. (2022). Feasibility of FreeSurfer Processing for T1-Weighted Brain Images of 5-Year-Olds: Semiautomated Protocol of FinnBrain Neuroimaging Lab. *Frontiers in Neuroscience*, *16*. https://doi.org/10.3389/fnins.2022.874062
